# Exploring the utility of Sentinel-2 MSI and Landsat 8 OLI in burned area mapping for a heterogenous savannah landscape

**DOI:** 10.1371/journal.pone.0232962

**Published:** 2020-05-27

**Authors:** Fiona Ngadze, Kudzai Shaun Mpakairi, Blessing Kavhu, Henry Ndaimani, Monalisa Shingirayi Maremba

**Affiliations:** 1 Allied Systems, Harare, Zimbabwe; 2 Geo-information and Earth Observation Centre, Department of Geography and Environmental Science, University of Zimbabwe, Mount Pleasant, Harare, Zimbabwe; 3 Zimbabwe Parks and Wildlife Management Authority, Harare, Zimbabwe; Potsdam Institute for Climate Impact Research, GERMANY

## Abstract

When wildfires are controlled, they are integral to the existence of savannah ecosystems and play an intrinsic role in maintaining their structure and function. Ample studies on wildfire detection and severity mapping are available but what remains a challenge is the accurate mapping of burnt areas in heterogenous landscapes. In this study, we tested which spectral bands contributed most to burnt area detection when using Sentinel-2 and Landsat 8 multispectral sensors in two study sites. Post-fire Sentinel 2A and Landsat 8 images were classified using the Random Forest (RF) classifier. We found out that, the NIR, Red, Red-edge and Blue spectral bands contributed most to burned area detection when using Landsat 8 and Sentinel 2A. We found out that, Landsat 8 had a higher classification accuracy (OA = 0.92, Kappa = 0.85 and TSS = 0.84)) in study site 1 as compared to Sentinel-2 (OA = 0.86, Kappa = 0.74 and TSS = 0.76). In study site 2, Sentinel-2 had a slightly higher classification accuracy (OA = 0.89, Kappa = 0.67 and TSS = 0.64) which was comparable to that of Landsat 8 (OA = 0.85, Kappa = 0.50 and TSS = 0.41). Our study adds rudimentary knowledge on the most reliable sensor allowing reliable estimation of burnt areas and improved post-fire ecological evaluations on ecosystem damage and carbon emission.

## 1. Introduction

Wildfires have been topical in most parts of the world, and recently got more attention following the California and Australian fires [1,2]. During the wildfire suppression paradigm, wildfires were not fully understood and were viewed as ecosystem disruptors [3]. To date, we know wildfires, when controlled are integral to the existence of savannah ecosystems and play an intrinsic role in maintaining their structure and function [4,5]. However, 340 million hectares burn annually and according to Andela and van der Werf [6], Africa contributes 70% of the global burnt area. Additionally, future projections indicate a possible increase in wildfire risk attributed from clearing of vegetation cover [7]. Although integral, wildfires are also contributing immensely to global carbon emissions [8], and their detection remains central to wildfire ecology.

There have been ample studies on wildfire detection and severity mapping with remote sensing (e.g. in Smith, *et al*. [9], Falkowski, *et al*. [10], Mohler and Goodin [11], Schepers, *et al*. [12] and Chuvieco, *et al*. [13]). As such, several sensors such as Advanced Very High Resolution Radiometric (AVHRR)[14], Advanced Space borne Thermal Emission and Reflection Radiometer (ASTER) [10] and Moderate Resolution Imaging Spectroradiometer (MODIS) [15] have been utilized. Of these sensors, MODIS has been frequently used due to its high temporal resolution which allows rapid detection of active-fires [16,17], allowing timely decisions to be made and reduce burn-date uncertainty [18,19]. However, MODIS has a coarse spatial resolution which makes detecting the spatial extent of smaller fires more difficult. Fortunately, the launch of Landsat-8 OLI (30m) and Sentinel-2 (10m) have allowed the use of sensors with a better spatial resolution [20].

The effectiveness of Landsat 8 OLI in detecting burned scars has been extensively explored due to its wide spatial coverage and free availability [21,22]. Landsat imagery has been widely utilized in detecting active fires and areas burned in several urban and rural environments [23–27]. Most of these studies make use of the shorter wavelength bands 4 and 7. However, Kumar and Roy [28] argue that the lack of middle-infrared bands in all Landsat sensors still poses a challenge in active fire mapping. Despite its reduced temporal resolution, Landsat imagery has proved to be both economic and reliable in detecting burned areas. However, the shorter revisit cycle (5 days) and high spatial resolution from Sentinel-2 multispectral instrument are better variables in burned area mapping [29]. Studies that have tested Sentinel-2 and its subsidiary Sentinel 2A indicate improved accuracy when Sentinel-2A are used for burned area detection and reforestation [29–31]. This is a result of Sentinel-2A’s near-infrared (NIR) and red-edge spectral bands. However, Sentinel-2’s high sensitivity to cloud cover [32] has been attributed as its main flaw in burned area detection. However, besides these setbacks, Sentinel-2 and Landsat 8 remain the widely used products in natural resources management [9,20,25,33,34].

Nevertheless, fewer studies have been done to compare the discrimination accuracy of burned and unburned areas between Sentinel-2A and Landsat 8 in the savannah ecosystem. Knowledge on the most reliable sensor allows for reliable estimation of burnt areas and improves burnt detection algorithms. Subsequently, this would also improve post-fire ecological evaluations on ecosystem damage and carbon emission. Notwithstanding their limitations, both products are cost-effective resources since they are freely available. Hence, we aimed to test which sensor performs best in detecting burnt areas in savannah ecosystems, which are greatly shaped by wildfires. We also intended to identify the band that contributed most in burnt area detection from both Sentinel-2 and Landsat 8 sensors. We hypothesized that wildfire detection capabilities for the two medium resolution sensors would be the same. The hypothesis was tested in two study sites in Zimbabwe with different vegetation types and terrain.

In developing countries such as Zimbabwe, wildfires have been reported to affect socio-economic aspects of the country’s development. These fires have been reported to be concentrated in the northern region of Zimbabwe [35,36] and accurately mapping these wildfires allows an understanding of their drivers. About the drivers, from Chinamatira, *et al*. [37] we know that arson and human negligence account for 86% and natural phenomena account for 14%. Patience Zisadza-Gandiwa, *et al*. [38] also reasoned that human activities such as animal poaching and land clearing for agricultural purposes are major drivers of wild fires.

## 2. Materials and methods

### 2.1. Study sites

**Fig 1:**
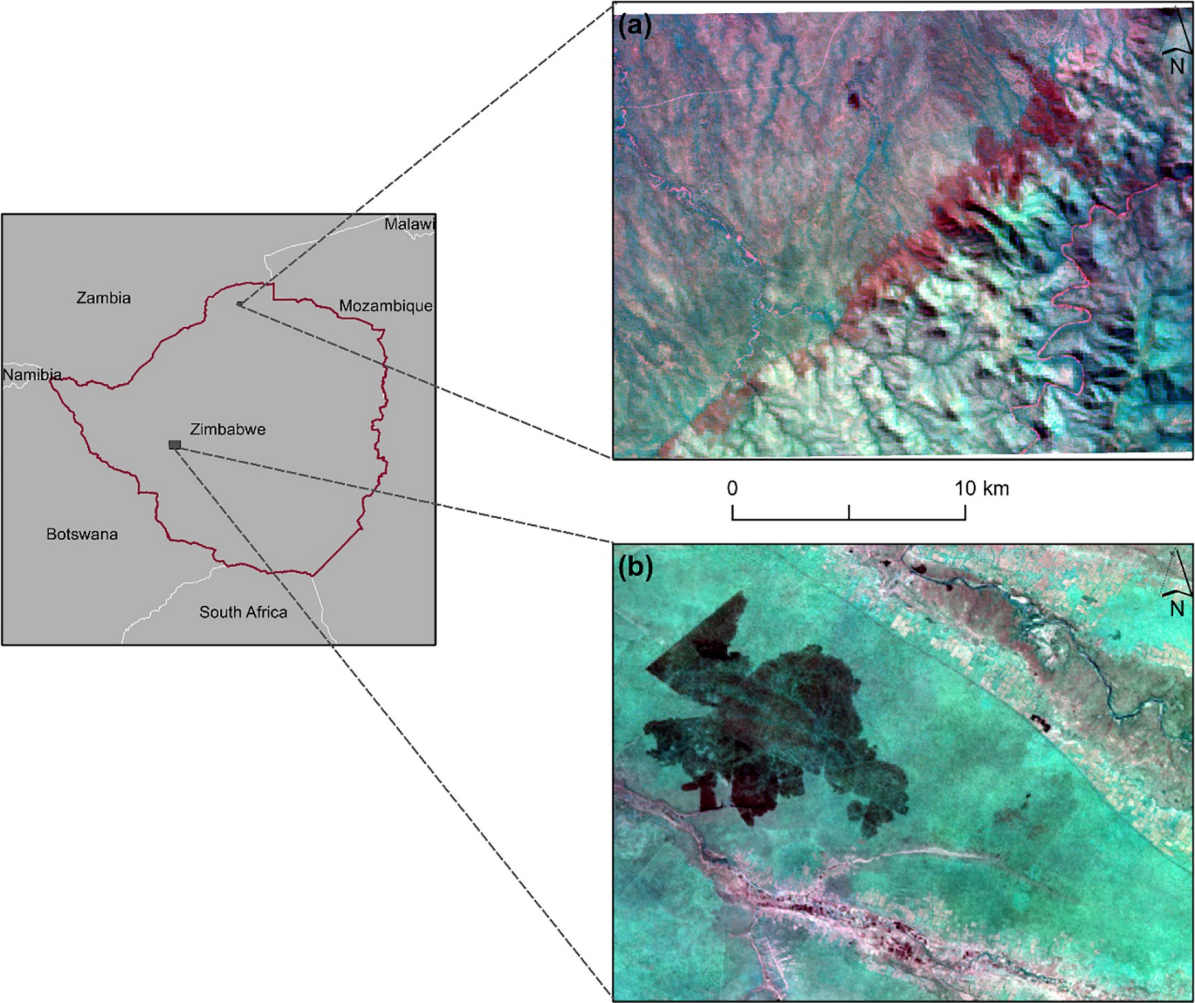
Location of the two sites used for this study. The study sites, (a) site 1 and (b) site 2, are overlaid on a composite image from Sentinel-2A imagery. The imagery was freely downloaded from http://earthexplorer.usgs.gov. The imagery is an RGB image with red = red band, green = water vapor band and blue = near infrared band. The burnt areas are the dark purple areas.

The study was conducted in two study sites that are located in the northwestern part of Zimbabwe (Fig 1). Study site 1 is part of Mana pools National Park. Study site 1 has rugged terrain and low elevation ranging between 600-900m. Study site 2 is part of Mbembesi State Forest and has flat terrain with elevation ranging from 1090-1100m.

The study sites are savannah environments and wildfire hotspots, hence their use in this study [35,36]. Wildfires are not permitted in both study sites with the exception of management fires. These management fires usually help in reopening fireguards and clearing moribund. More information on the study sites is shown in Table 1.

**Table 1:** A description of some of the biophysical characteristics of the two study sites that were used in this study.

Study Site	Location	Annual Temperature	Annual Precipitation	Land-Use Type	Date of Burn	Date of image Acquisition and Scene for Sentinel2A and Landsat 8
Site 1	29° 5' 22.3008'' °E	17.5°C-33°C	450–650 mm [39]	Safari area (Sparsely vegetated, with setaria grasslands)	2017-07-01–2017-07-02	2017-07-24 L1C_T35LQC
	16° 3' 44.8164'' S					2017-07-10 LC08_L1TP_171071
Site 2	28° 12' 46.908'' E	17.5°C—33°C	500mm- 750 mm [40].	Forestry area (Predominantly miombo woodlands)	2017-09-15–2017-09-17	2017-09-22 L1C_T35KPU
	19° 17' 33.612'' S					2017-09-28 LC08_L1TP_171073

### 2.2. Permits

Data for the study sites were freely available online, hence no permits were required.

### 2.3. Data acquisition and pre-processing

For each study site, two cloud-free satellite images for Sentinel 2A and Landsat 8 OLI were downloaded from https://earthexplorer.usgs.gov/ (Accessed 18 June 2018). The images used in this study were those available and acquired closest to the date of burn. For both study sites, the images downloaded were post-fire images. A description of the spectral bands that make up Sentinel 2A and Landsat 8 OLI is shown in Table 2.

**Table 2:** Spectral bands present in both Sentinel 2A and Landsat 8 OLI.

Landsat-8 OLI			Sentinel 2A		
Spectral bands	Central wavelength (μm)	Resolution (m)	Spectral bands	Central wavelength (μm)	Resolution (m)
B1 –Coastal aerosol	0.443	30	B1 –Coastal aerosol	0.443	60
B2 –Blue (B)	0.482	30	B2 –B	0.494	10
B3 –Green (G)	0.561	30	B3 –G	0.56	10
B4 –Red (R)	0.655	30	B4 –R	0.665	10
-	-	-	B5 –Red edge 1	0.704	20
-	-	-	B6 –Red edge 2	0.74	20
-	-	-	B7 –Red edge 3	0.78	20
B5 –Near infrared (NIR)	0.865	30	B8 –NIR	0.843	10
			B8A –NIR narrow	0.864	20
			B9 –Water vapor	0.944	60
B9 –Cirrus	1.373		B10 –SWIR Cirrus	1.375	60
B6 –Shortwave infrared (SWIR1)	1.609	30	B11 –SWIR1	1.612	20
B7 –Shortwave infrared (SWIR2)	2.201	30	B12 –SWIR2	2.194	20
B8 –Panchromatic	0.59	15	-	-	-
B10 –Thermal Infrared 1	10.895	100	-	-	-
B11 –Thermal Infrared 2	12.005	100	-	-	-

Prior to their use in any analysis, we atmospherically corrected both images. Sentinel-2 data were atmospherically corrected in SNAP [41] using the sen2cor tool [42]. Landsat 8 data were atmospherically corrected in ENVI [43] using the Fast Line-of-sight Atmospheric Analysis of Hypercubes (FLAASH) [44] and the Thermal Atmospheric Correction tool for multispectral data and thermal data respectively. The algorithms minimize atmospheric effects such as scattering, thus improving the reflectance of each spectral band. We used this method following Chrysafis, *et al*. [45] and Fernández-Manso, Fernández-Manso and Quintano [30]. We did not geometrically correct Sentinel 2A or Landsat 8 OLI because these images are provided geometrically corrected [46–48].

### 2.4. Classification

To test our hypothesis, we classified our post-fire Sentinel 2A and Landsat 8 images using the Random Forest (RF) classifier in R [49] within the caret package[50]. RF is a classification and regression ensemble algorithm that uses machine learning techniques to build a forest from decision trees [51]. The RF algorithm can handle large datasets with high dimensionality and will still not overfit [52]. The RF algorithms is optimized based on the number of regression trees grown (n_tree_) and the number of predictors used (mtry) at each split as it creates a new tree (node) [53]. The mtry used at each node affects the accuracy of a tree grown and increasing the n_tree_ increases the models performance [54]. To determine the m_try and_ n_trees_ to use that would optimize model performance we used tenfold cross validation repeated thrice from a range of 1–6 m_try_ and 50–1500 n_trees_. The model with the lowest RMSE was selected as the final model.

Classification was done using the blue, green, red, red-edge, narrow near infrared (NIRn), shortwave infrared 1(SWIR 1) and, shortwave infrared 2 (SWIR 2) spectral bands for Sentinel-2A. For Landsat 8, the blue, green, red, near infrared (NIR), shortwave infrared 1 (SWIR 1), shortwave infrared 2 (SWIR 2), thermal infrared 1 (TIR 1) and thermal infrared 2 (TIR 2) spectral bands were used for classification. The water vapor, panchromatic and coastal aerosol spectral bands were excluded because of their irrelevance to burnt area mapping.

Point data for burned and unburned areas were used to train the RF algorithm. Burned areas were trained using Visible Infrared Imaging Radiometer Suite (VIIRS) active fire data downloaded from https://firms.modaps.eosdis.nasa.gov/ (accessed 10/07/2018). VIIRS data is provided at a resolution of 375m. The point data were converted to raster files with a cell size of 375m and the raster files were re-sampled to 20m and 30m to match Sentinel-2 and Landsat 8 imagery respectively. We then extracted the central coordinates of each re-sampled raster pixel to train burned areas in our classification. Unburned areas were trained using photo-interpreted points from post fire imagery. The training points for unburned areas were randomly generated.

Random forest classification performance was measured using the overall accuracy (OA), Cohen’s Kappa (Kappa) and True Skill Statistics (TSS). These measures have been widely used with machine learning algorithms to measure how well an algorithm discriminates between one land cover and the other [55]. The OA and Kappa were calculated through bootstrapping the training data 25times. TSS was calculated from the specificity and sensitivity derived from the 2 X 2 contingency table matrix. OA, Kappa and TSS range from 0 to 1. 0 representing strong disagreement and 1 representing strong agreement. To test the difference in the classification accuracy of the two sensors, a T test was used following Dube, *et al*. [56].

### 2.5. Variable contribution

The decrease in node impurities measured by the Gini index was used to find the spectral band that contributed most to the overall classification for both sensors. The metric measures how well a variable contributes to node homogeneity. An important variable would be frequently used to create nodes hence giving a higher decrease in node impurities [57]. A description of how the Gini index is implemented in R is explained in Cutler, Cutler and Stevens [53]. The spectral band with the highest Gini index was considered the most important compared to the other spectral bands in all the study sites for both sensors.

## 3. Results

**Fig 2:**
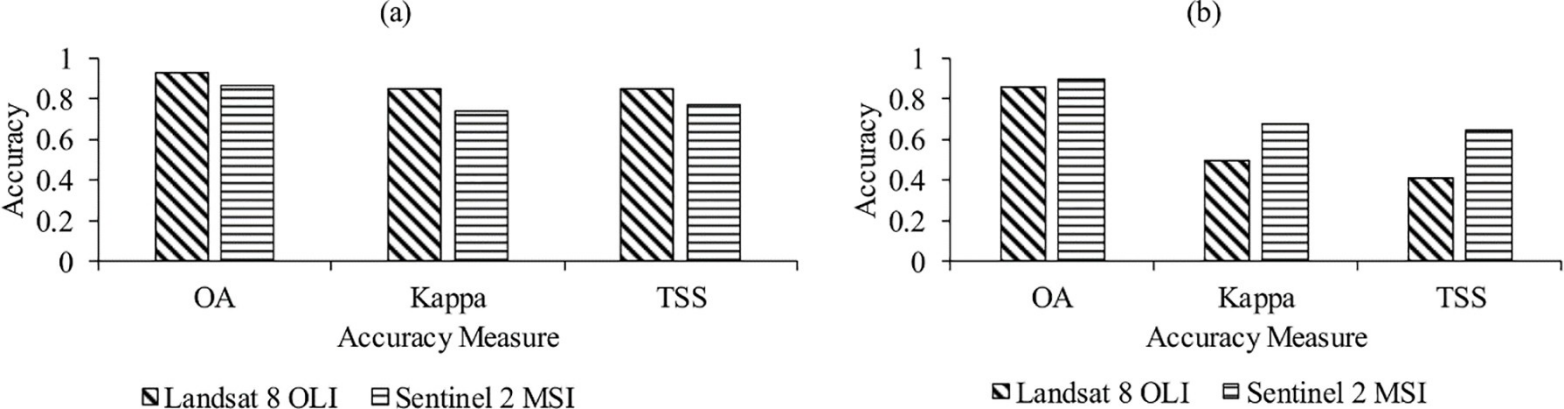
Classification accuracy for Sentinel -2A and Landsat 8 OLI in (a) study site 1 and (b) study site 2 using the overall accuracy (OA), Cohen’s Kappa (Kappa) and True Skill Statistics (TSS).

In the two study sites that our hypothesis was tested, our results show that both Landsat 8 and Sentinel 2A had high burned area detection (OA >0.86, Kappa >0.5 and TSS >0.41) (Fig 2). In addition, the classification output in the two study sites was significantly different for Landsat 8 and Sentinel-2 (t = 58.333, p-value = 0.0000111). Landsat 8 had a higher classification accuracy (OA = 0.92, Kappa = 0.85 and TSS = 0.84)) in study site 1 as compared to Sentinel-2 (OA = 0.86, Kappa = 0.74 and TSS = 0.76). In study site 2, Sentinel-2 had a slightly higher classification accuracy (OA = 0.89, Kappa = 0.67 and TSS = 0.64) which was comparable to that of Landsat 8 (OA = 0.85, Kappa = 0.50 and TSS = 0.41).

**Fig 3:**
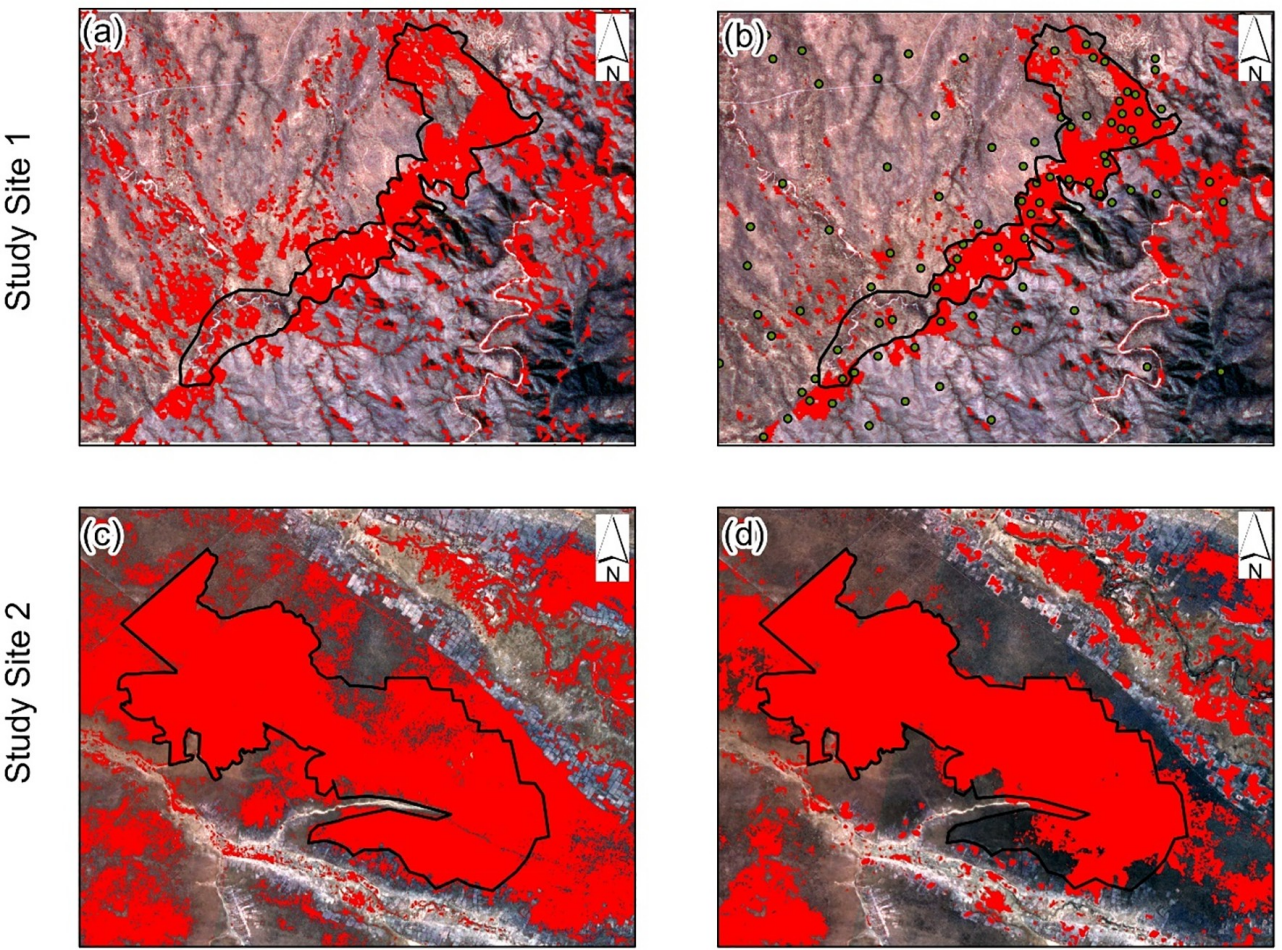
Burned (red) areas classified using the random forest algorithm with Sentinel-2 (a and c) and Landsat 8 imagery (b and d). The burnt areas are overlaid on Sentinel-2 natural color imagery. The black outline shows the field measured burned area. The imagery was freely downloaded from http://earthexplorer.usgs.gov.

When compared to field measurements, observation of the classified images shows that in both study sites, Sentinel-2 imagery overestimated the burnt area as compared to Landsat 8 (Fig 3).

**Fig 4:**
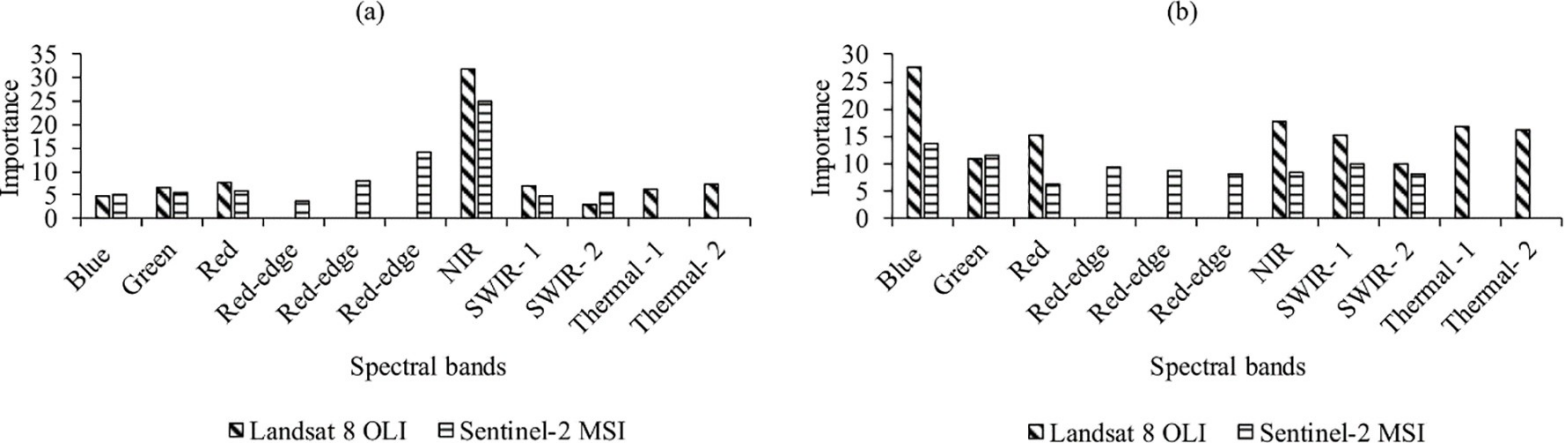
The contribution of each spectral band to burn area mapping with Sentinel -2A and Landsat 8 OLI in (a) study site 1 and (b) study site 2.

When detecting burned areas using Landsat 8, the NIR, Red and Blue spectral bands contributed most to overall burnt area detection in both study sites. The shortwave infrared 2 spectral band was the least contributing band when using Landsat 8 in both study sites (Fig 4). The NIRn and Blue spectral bands contributed most to overall classification using Sentinel-2 in study site 1 and 2 respectively. In addition, the red-edge and the red band from Sentinel-2 contributed least to overall classification in study site 1 and 2 respectively. Spectral bands that contributed the most were similar in study site 1 and 2 regardless of the sensor used.

## 4. Discussion

In this study, we aimed to find which spectral bands contributed most to burned area detection when using Sentinel-2A MSI and Landsat-8 OLI. We also aimed to find which sensor had a higher classification accuracy in detecting burnt areas. These aims as well as the working hypothesis were answered by our results. Our results confirm and dispute previous research that has been done on burnt area mapping in savannah environments.

Both Sentinel-2A and Landsat 8 had high classification accuracy in classifying burned areas (OA >0.86, Kappa >0.5 and TSS >0.41) in our two study sites. In study site 1, Landsat 8 had a higher classification accuracy (OA = 0.92, Kappa = 0.85 and TSS = 0.84) than Sentinel- 2A (OA = 0.86, Kappa = 0.74 and TSS = 0.76). We found out that the NIR, red and red-edge spectral bands from Landsat 8 and Sentinel-2A contributed significantly to burned area mapping, in study site 1. The NIR and Red spectral bands were from Landsat 8 and the NIR and red-edge spectral bands were from Sentinel-2A. Depending on wildfire severity, wildfires can completely burn or damage vegetation but in either way this affects vegetation reflectance [22,58]. When vegetation is damaged, this affects the leaf structure, composition and functioning reducing reflectance in the NIR spectral region [9,11,12]. Vegetation reflectance in the NIR strongly depends on the spongy mesophyll [59,60] and fire related stress affects the mesophyll thus reducing reflectance in the NIR [61]. Mohler and Goodin [11] observed a drop in NIR spectral reflectance after burning. As a result of these noticeable fluxes in vegetation reflectance within the NIR spectral region, this is why vegetation spectral indices such as Normalized Difference Vegetation Index (NDVI) [62], Char Soil Index (CSI) [63] and Normalized Burn Ratio (NBR) [64] have been used extensively for burned vegetation monitoring [13].

Chlorophyll present in healthy vegetation induces reflectance mainly in the red and red-edge spectral regions. When wildfires damage vegetation and consequently the photosynthetic cells where chlorophyll is present, this affects the chlorophyll content in plants and reduces reflectance in the red spectral region [30,31]. Equally important, the red-edge spectral region is sensitive to subtle changes in chlorophyll [34]. Changes to chlorophyll can either broaden or shorten its reflectance between 670-680nm [59]. Wildfire related damage to vegetation causes vegetation reflectance to move towards the shorter wavelength (i.e. shortening the 670-680nm region) [59,65]. These changes are detectable in the red-edge spectral region hence its enhanced contribution in mapping burned areas in our study. Our results are synchronous to studies that have used the red-edge spectral band or its related spectral indices and reported high accuracy than the conventional bands [59,65,66].

In study site 2, our results illustrate that Sentinel-2A performed better (OA = 0.89, Kappa = 0.67 and TSS = 0.64) in burned area mapping however the results were comparable to those of Landsat 8 (OA = 0.85, Kappa = 0.50 and TSS = 0.41). The blue spectral band from Sentinel 2A and Landsat 8 contributed most to burnt area mapping in study site 2. Healthy vegetation is known to absorb blue wavelength through the presence of carotenoids, xanthophyll or chlorophyll [67]. Since wildfires affect leaf structure and photosynthetic ability, this also decreases the green leaf pigment (chlorophyll) and increases the brown-yellow pigment (carotenoids, pheophytin and xanthophyll) [68]. Increase in the brown-yellow pigment as a result of increased carotenoids, pheophytin and xanthophyll causes an increase in vegetation reflectance in the blue spectral band [25,33,69]. Observations made by Pleniou and Koutsias [70] also show that burned areas have a higher reflectance in the blue spectral band when compared to vegetated areas. Increased reflectance in the blue spectral region has also been observed in vegetation undergoing senescence [71,72]. In addition, the contribution of the blue spectral region could be plausibly due to the time of burn since the vegetation in study site 2 is deciduous and chlorophyll content was low further increasing reflectance in the blue spectral region. Our results on the performance of the blue spectral band are novel and dispute earlier studies [73] that discredit the blue spectral band in burn area mapping.

Lastly, the difference in performance between Landsat-8 and Sentinel-2 could possibly be related to the date of image acquisition. We observed that, imagery that was acquired closet to the date of burn had high classification accuracy. The detectability of a fire scar with remote sensing is primarily based on the burn severity and vegetation type [74–76]. Since wildfires can positively influence vegetation regeneration [74,77] this can also negatively affect spectral separability of burned and unburned areas [78] especially when the date of image acquisition is further away from the date of burn. The overestimation of Sentinel-2 imagery in study site 1 could be attributed to the fact that, Sentinel-2 imagery used was acquired three weeks after the wildfire. In study site 2, although Landsat 8 imagery was acquired twelve days after the date of burn, it had high classification accuracy and did not overestimate burn areas when visually compared to Sentinel-2A. These observations could possibly mean that although Landsat 8 has a low spatial resolution as compared to Sentinel-2, but it can still be utilized in burned area mapping even when the images are acquired further away from the date of burn.

Studies that have compared Landsat 8 and Sentinel-2A have usually focused on their classification accuracy in land cover mapping [79], vegetation health [59] and burn severity [30]. Of these studies only a handful focused on the contribution of each spectral region in the mapping process. Although Sentinel-2A has a higher spatial resolution (10m) than Landsat 8 (30m), the inclusion of the thermal band in Landsat 8 allows for better burnt area detection and mapping [80,81]. The thermal band has been used to detect radiance in and around burned areas [82,83] during a satellite overpass. Consequently, in study site 1, the thermal band was the third most important contributor to Landsat 8 burned area mapping. However, the band has some calibration issues that need to be addressed to allow accurate use in burned area mapping [84]. The high accuracy exhibited by Landsat 8 as compared to Sentinel -2A in burnt area mapping, is coherent to observation made by Mallinis, Mitsopoulos and Chrysafi [22]. Mallinis, Mitsopoulos and Chrysafi [22] observed that Landsat 8 spectral indices had a higher spectral separability than Sentinel-2A in burned area mapping. Hence our study is reaffirming in that it is amongst the few studies that have tested how spectral regions from Sentinel 2A and Landsat 8 contribute to burned area mapping, in study sites with different land-uses.

We recommend that future studies on burnt area mapping should include the blue spectral band in burned area mapping. Schepers, Haest, Veraverbeke, Spanhove, Borre and Goossens [12] recommended that using these spectral regions could improve our insight pertaining to burned area mapping. Additionally, we also recommend that more studies be conducted that investigate the fusion and continued use of Sentinel-2 and Landsat 8 imagery in burn area mapping.

## 5. Conclusion

Knowledge on the most reliable sensor allows for reliable estimation of burnt areas and improves post-fire ecological evaluations on ecosystem damage and carbon emission. Hence, in this study, we aimed to find which spectral bands contributed most to burned area detection when using Sentinel-2A and Landsat 8 OLI. We also aimed to find which sensor had higher classification accuracy in detecting burnt areas. Our findings suggest that, indeed the near infrared, red and red-edge spectral regions are essential in burn area mapping. We also propose the use of the blue spectral region in detecting burned areas since it contributed considerably to burn area mapping as compared to commonly used spectral regions such as the shortwave infrared. Whilst the date of image acquisition affects burn detection and mapping, our results also show that although Landsat 8 OLI has a low spatial and temporal resolution as compared to Sentinel-2, it can still be utilized in burn area mapping. Considering the recent increase in burn areas globally, these results provide rudimentary information relevant for accurate and timely burn area detection as well as post-fire damage assessments.
